# *Novel* variant in *MYH9* in a child with proteinuria and thrombocytopenia: a case report and literature review

**DOI:** 10.3389/fped.2025.1502727

**Published:** 2025-05-09

**Authors:** Dan-Feng Xie, Lin Zhu, Xiao-Meng Wang, Yun Li, Ping Zhou

**Affiliations:** ^1^ Department of Pediatric Nephrology, Allergy, and Rheumatology, Sichuan Provincial Women’s and Children’s Hospital, The Affiliated Women’s and Children’s Hospital of Chengdu Medical College, Chengdu, Sichuan, China; ^2^ Sichuan Clinical Research Center for Pediatric Nephrology, Chengdu, Sichuan, China

**Keywords:** proteinuria, thrombocytopenia, liver dysfunction, FSGS, *MYH9*-RD, child, case report

## Abstract

There is a lack of awareness of the diagnosis and treatment of *MYH9*-related disorder (*MYH9*-RD), which is an autosomal dominant disease with heterogeneous clinical manifestations. We summarized the clinical phenotype and reported a *novel* variant in *MYH9* in a child with focal segmental glomerulosclerosis (FSGS) and reviewed the relevant literature to better understand *MYH9*-RD. Unlike previous cases, this patient exhibited IgA deposition in the mesangial region, suggesting need for further investigation into prognostic and therapeutic significance of this finding. To reduce the risk of *MYH9*-RD misdiagnosis, we recommend assessing mean platelet diameter and granulocyte inclusions in patients with unexplained proteinuria and refractory thrombocytopenia.

## Introduction

*MYH9*-RD is a group of autosomal dominant disorders caused by variants of the *MYH9* gene, which encodes nonmuscle myosin heavy chain IIA (NMMHC-IIA). The incidence of *MYH9*-RD is 1–9/1 000 000 (1) and 20%–35% of *MYH9*-RD cases are sporadic (2). *MYH9*-RD features macrothrombocytopenia and granulocyte inclusions with or without nephropathy, sensorineural hearing loss, cataracts and elevated serum aminotransferase. NMMHC-IIA is expressed in most cells and tissues including platelets, leukocytes, kidneys and cochlea. NMMHC-IIA is part of the myosin superfamily, and three isoforms are present in humans. Tissue-specific distribution of the isoforms may account for the diverse clinical manifestations of *MYH9*-RD (3, 4). To date, several *MYH9* variants have been implicated in *MYH9*-RD. Renal involvement is reported in 30%–70% of patients. However, renal pathological findings in *MYH9*-RD patients have been reported infrequently because of the rarity of *MYH9*-RD, and thrombocytopenia. Patients with *MYH9*-RD may be misdiagnosed within idiopathic thrombocytopenic purpura, IgA nephropathy or Alport syndrome. Misdiagnosis can result in potentially harmful treatments, such as glucocorticoids, immunosuppressive agents and splenectomy. In this case report, we analyzed the clinical features, laboratory results, pathological biopsy, immunofluorescence and gene sequencing of a child with *MYH9*-RD. To better understand *MYH9*-RD, especially kidney injury, we also reviewed the relevant medical literature.

## Case presentation

### Medical history summary

*Chief complaints:* An 8-year-old boy was admitted to hospital due to abnormal liver function for 7 years and proteinuria for 3 years.

A comprehensive timeline of the diagnosis and treatment of *MYH9*-RD is provided in Table 1. Aside from ear secretions, hearing loss, and abnormal liver function, the child also had proteinuria, thrombocytopenia, and no bleeding symptoms. At age 11 months, he was diagnosed with patent ductus arteriosus, thrombocytopenia and liver dysfunction. At age 4 years, he was diagnosed with bilateral tonsillitis, adenoid hypertrophy, left secretory otitis media, mild bilateral mixed hearing loss, thrombocytopenia and liver dysfunction. At age 5 years, he was diagnosed with binaural secretory otitis media, binaural mixed moderate hearing loss, thrombocytopenia, liver dysfunction and isolated albuminuria. He was treated with double tympanic membrane catheterization to improve hearing loss, and polyene phosphatidylcholine and glycyrrhizin to reduce liver cell damage for 1 week. Liver function showed that decreased transaminase levels, but total bile acid did not improve. At age 8 years, he was admitted to Sichuan Provincial Maternity and Child Health Care Hospital due to abnormal liver function and proteinuria.

**Table 1:** Timeline of diagnosis and treatment for the pediatric patient with MYH9-RD.

Age	Diagnosis	Clinical treatment
<1 year	Patent ductus arteriosus	Arterial catheter occlusion
	Thrombocytopenia	Hepatoprotective drugs
	Liver dysfunction	
4 year	Bilateral tonsillitis	Hepatoprotective drugs
	Adenoid hypertrophy	
	Left secretory otitis media	
	Mild mixed bilateral hearing loss	
	Thrombocytopenia	
	Liver dysfunction	
5 year	Moderate binaural mixed hearing loss	Ear tympanic membrane tube placement
	Liver dysfunction	Hepatoprotective drugs
	Isolated proteinuria	
8 year	*MYH9*-RD	Oral Benazepril Hydrochloride
		Hepatoprotective drugs

The patient had a history of patent ductus arteriosus and previously underwent transcatheter closure at 11 months of age. The patient had no history of hemorrhagic disease. The patient's parents are not related. So far, their liver functions and urine tests are normal.

Physical examination revealed normal blood pressure, normal appearance, and neuromotor development without backwardness; no edema, rash, or ecchymosis; superficial lymph nodes without enlargement; bilateral lung physical examination showed no abnormalities; liver and spleen palpation without enlargement; and no nervous system abnormalities.

### Routine clinical examinations

A routine blood test revealed a white blood cell count of 4.62 × 10^9^/L, neutrophil percentage 46%, lymphocyte percentage 43%, hemoglobin concentration 132 g/L, total platelet count 106 × 10^9^/L, and average platelet volume 14.0 fL. Urine protein to creatinine ratio (Up/Ucr) 218.45 mg/g; urinary tetraprotein test: microalbumin 392.6 mg/L; transferrin 23.61 mg/L; β2 microglobulin or α1 microglobulin: normal; 24-h urinary protein concentration 547.5 mg/d. The 24-h urinary calcium and copper were normal. Liver function parameters were: alanine aminotransferase 111.0 U/L, aspartate aminotransferase 188.0 U/L, alkaline phosphatase 544.0 U/L, γ-glutamyl transferase 86.7 U/L, and total bile acid 17.7 μmol/L. Blood lipid and lipoprotein levels were normal. There were no abnormalities in renal function or electrolytes. Coagulation function was normal; hepatitis B virus DNA was negative, hepatitis A and E antibodies were negative, hepatitis C core antigen was negative, hepatitis C antibody was negative, tuberculosis antibody was negative, Epstein–Barr virus antibody was negative, and syphilis and AIDS related screening were all negative. Quantitative determination of immunoglobulin subclass was normal, rheumatoid factor was negative, antinuclear antibody was negative. The serum lead level was 19.8 μg/L. Laboratory indicators are presented in Table 2.

**Table 2:** Important laboratory indicators.

Laboratory examination item	Result	Reference interval for normal values
WBC (×10^9^/L)	4.62	4–10
HGB (g/L)	132	110–130
PLT (×10^9^/L)	106	100–300
Average platelet volume (fL)	14.0	9–13
ALT (U/L)	111	5–40
AST (U/L)	188	5–40
Blood lipid and lipoprotein	Nomal	/
Up/Ucr (mg/g)	218.45	<30
24-h urinary protein concentration (mg/d)	547.5	<150
Renal function	Nomal	/
Serum electrolytes	Nomal	/
Coagulation function	Nomal	/
Tuberculosis antibody	(−)	(−)
Hepatitis virus (A, B, C, E)	(−)	(−)
Syphilis and AIDS related screening	(−)	(−)
Epstein–Barr virus antibody	(−)	(−)
24-h urinary calcium (mmol/24 h)	5.3	2.7–7.5
24-h urinary copper (μg/d)	21.8	15–30
Immunoglobulin subclass	Nomal	/
RF	(−)	(−)
Antinuclear antibody	(−)	(−)
Serum lead (μg/L)	19.8	0–99

WBC, white blood cell count; HGB, hemoglobin concentration; PLT, platelet count; ALT, alanine aminotransferase; AST, aspartate aminotransferase; Up/Ucr, Urine protein to creatinine ratio; RF, rheumatoid factor.

The patient and his parents underwent a peripheral blood smear examination. After Wright staining, macrothrombocytopenia were found in the patient's peripheral blood smear , and no abnormal granulocyte inclusions were found. Medium and large (4–7 μm) platelets accounted for 40%–65%, and giant platelets (>7 μm) accounted for 9%–15%. The peripheral blood smears of the parents were normal. Bone marrow smear revealed active hyperplasia, and the proportion and morphology of granulocytes, erythroid cells and lymphocytes was normal. The entire bone marrow smear showed 23 megakaryocytes, clusters and scattered platelets, as well as large and giant platelets.Chest x-ray revealed no abnormalities. Upper and middle abdominal magnetic resonance imaging showed that the liver, gallbladder, pancreas, spleen and kidneys were normal. Urinary ultrasound revealed no abnormalities in either kidneys or ureters.

Pure tone audiometry and acoustic impedance tests revealed moderate binaural mixed hearing loss. Visual acuity, lens and fundus examination were normal.

### Subsequent laboratory tests

The pathological diagnosis of liver biopsy indicated mild liver injury. Inflammatory necrosis activity in and around the portal area showed mild detrital necrosis. Intralobular inflammatory necrosis activity was characterized by focal necrosis and the presence of eosinophilic bodies. The fibrosis stage indicated fibrotic enlargement in the portal area, corresponding to a modified Scheuer score of G2S1.

**Figure 1:**
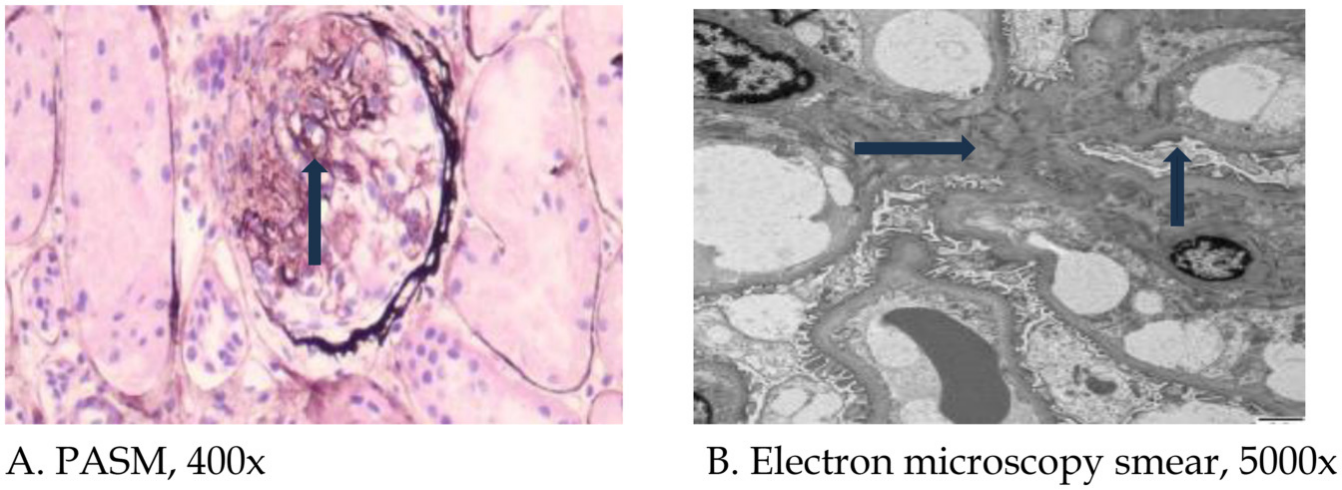
Pathological light and electron microscopy images of renal biopsy. **(A)** Arrow indicates focal segmental glomerulosclerotic changes (PASM, 400×); **(B)** Blue arrow indicates widening of the mesangial region, and black arrow indicates foot process fusion (5,000×). PASM: periodic acid-silver methenamine.

*Pathological findings of renal biopsy*: Specimens were stained with hematoxylin and eosin, PAS, periodic acid methenamine silver (PAM) or Masson's trichrome stains. Paraffin sections were stained with periodic acid-silver methenamine (PASM), and ten glomeruli were examined. Among these ten glomeruli, no glomerulosclerosis was observed; however, segmental sclerosis was identified in two glomeruli (Figure 1A). There was no significant thickening of the basement membrane, no evidence of eosinophilic deposits in the subepithelial and subendothelial layers, no noticeable proliferation of parietal epithelial cells, and no formation of crescents. Additionally, there was granular degeneration of renal tubular epithelial cells without significant atrophy, no apparent infiltration of lymphocytes or monocytes in the renal interstitium, and no substantial changes in the walls of small arteries. Immunofluorescence revealed IgA deposits in the form of dots along the mesangial region, complement component C3 (−), C1q (−), Fibrinogen (FIB) (−), albumin (−), normal α1-positive controls, and normal type IV collagen α3-chain and α5-chain. Ultrastructural results (Figure 1B) revealed that the thickness of the glomerular basement membrane was 180–380 nm, the foot process was segmentally fused, and a few electron-dense deposits were observed in individual mesangial areas.Pathological diagnosis revealed focal segmental glomerulosclerosis (FSGS), and immunofluorescence indicated the presence of IgA deposition.

**Figure 2:**
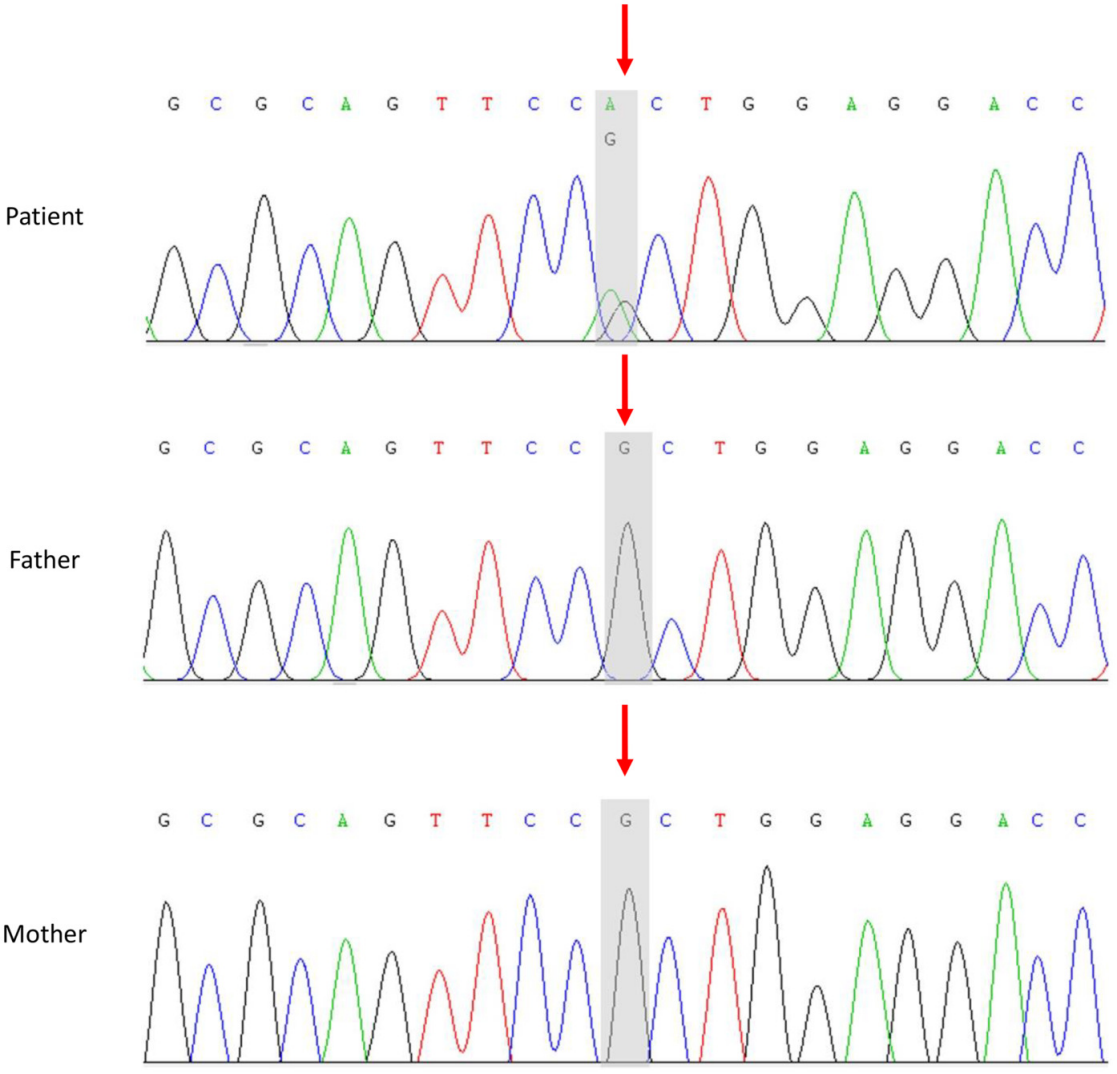
Sanger sequencing of the *MYH9* gene in the patient and his parents. In the patient, exon 20 of the *MYH9* gene contained a heterozygous missense variant, NM_002473.6: exon 20: c.2440C>T (p. Arg814Trp). The parents of the child had a normal wild type.

Several factors led us to suspect that this child might have a genetic disorder, including the young age at disease onset, multiple systems involved, an increase in platelet volume, neutrophil inclusion bodies, and the absence of specific lesions on liver and kidney biopsy. With the informed consent of his parents, peripheral blood samples were collected from the patient and his parents, and whole-exome analysis was conducted. *MYH9* gene exon 20 NM_002473.6: exon20: c.2440C>T (p. Arg814Trp) heterozygous base missense variant was detected in the patient, but the genotype of his parents was normal (Figure 2).

**Figure 3:**
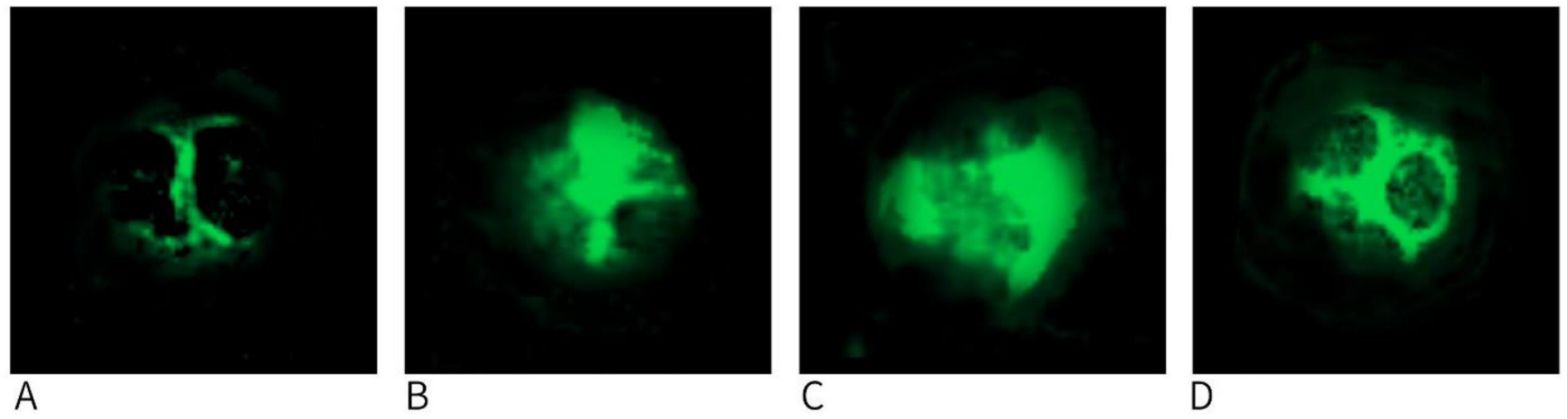
Immunofluorescence detection of nonmuscle myosin heavy chain IIA in neutrophils from the patient, his parents, and normal children. **(A)** Representative image (1,100× magnification) of a type III neutrophil inclusion body from the patient; **(B)** IF pattern in the father; **(C)** IF pattern in the mother; **(D)** IF pattern in the child without thrombocytopenia or *MYH9*-RD. IF: Immunofluorescence.

In accordance with the American College of Medical Genetics and Genomics (ACMG)’ guidelines, this locus may contain a pathogenic variant that has not yet been described (5). Considering the potential pathogenic nature of this variant and the absence of abnormal inclusion bodies in neutrophils after Wright's staining of peripheral blood smears under light microscopy, we conducted immunofluorescence assays to detect NMMHC-IIA in neutrophils from three family members, among whom the patient was included, and one normal control child. A fluorescence microscope (BX60; Olympus) with an excitation wavelength of 490 nm was used for observation. NMMHC-IIA in the cytoplasm of neutrophils from the normal control and parents of the patient showed a dispersed and homogeneous distribution, without green fluorescence aggregation. Dotted type III inclusions were scattered in the cytoplasm of neutrophils in the patient (Figure 3). This confirmed the diagnosis of *MYH9*-RD in the patient.

## Final diagnosis

A diagnosis of *MYH9*-RD was finally made based on the clinical phenotype, cell morphology, neutrophil immunofluorescence detection of NMMHC-IIA, and genetic screening. Based on the screening of the *MYH9* gene and the lack of a relevant family history, we concluded that this was a sporadic case. It took 7 years from the onset of illness to the time of diagnosis.

## Treatment

A compound glycyrrhizin (25 mg taken orally three times a day) and benazepril hydrochloride (5 mg taken orally once a day) regimen was administered to the patient for one year. No adverse drug reactions occurred.

## Outcome and follow up

Upon re-examination, serum transaminase content decreased to ∼100 U/L, Up/Ucr decreased to 113.20 mg/g, total platelet count was 129 × 10^9^/L, and his renal function remained at normal levels. There was no aggravation of his hearing impairment.

## Discussion

*MYH9*-RD is a rare monogenic inherited disease that has been reported in different races (6). Clinical features include hematological features that appear at birth (macrothrombocytopenia and granulocyte inclusions), and involvement of one or more organs such as the eyes, ears, kidneys and liver. *MYH9*-RD is caused by variants of the *MYH9* gene. The *MYH9* gene is located on chromosome 22q11.2 and it encodes NMMHC-IIA. NMMHC-IIA is a ubiquitous hexameric motor protein associated with cell movement, morphological maintenance and cell division (1, 7). There are 41 exons in the *MYH9* gene, including the N-terminal globular head (exons 2–19 coding region), neck (exon 20 coding region), tail coil domain (exons 21–40 coding region) and C-terminal nonspiral tail (exon 41 coding region). The first exon is not translated. As of July 19, 2023, the human gene variant database (HGMD, Professional Edition) listed 219 *MYH9* gene variants related to *MYH9*-RD, mainly missense variants (79.4%), followed by frameshift variants (7.8%), small in-frame deletions/insertions, nonsense variants, synonymous variants, and others. Among the *MYH9* pathogenic gene variants, 27.9% were in the head region, 2.7% in the neck region, 58.9% in the tail coiled coil domain, and 10.5% in the C-terminal nonhelical tail. S96 and R702 located in the head domain of NMMHC-II, as well as R1165, D1424 and E1841 located in the tail region of NMMHC-IIA, are the most common variant sites, accounting for ∼80% of all cases (8, 9). There are also cases of somatic or germ cell chimerism (10). In this study, we identify a *MYH9* gene variant in the neck region, which has not been previously reported in the literature.

It is important to highlight that the patient took 7 years to receive a diagnosis after the onset of symptoms. Timely and precise diagnosis of *MYH9*-RD can help avoid unnecessary treatments and mitigate delays in care. Patients with kidney issues should be tested for *MYH9*-RD if they present with unexplained macrothrombocytopenia, particularly if they have lesions in the eyes, ears, or live. There is a lack of consensus on the clinical diagnostic criteria for *MYH9*-RD. A mean platelet diameter greater than 3.7 μm and/or more than 40% of platelets exceeding 3.9 μm (approximately half the diameter of red blood cells) are highly sensitive and specific indicators for differentiating *MYH9*-RD from other hereditary or acquired forms of thrombocytopenia (11). An immunofluorescence assay that detects abnormal NMMHC-IIA localization in neutrophils has been validated as a diagnostic tool for *MYH9*-RD (12). The granulocyte inclusions in *MYH9*-RD patients can be categorized into three types based on their distribution: type I includes one or two large round or oval inclusions, type II consists of 3–20 dot-like inclusions smaller than 1 μm, and type III features scattered spotty inclusions in the cytoplasm. Saposnik et al. *(*13) later noted that the distribution of these inclusions correlates with the location of *MYH9* gene variants: type I inclusions are primarily linked to variants in exons 39, 40, and 41, while type II and III inclusions are often associated with variants in exons 2–31; variants in exons 32 and 33 can result in any type of inclusion. Wright's staining allows for easy identification of type I inclusions in peripheral blood smears, but type II and III inclusions are harder to detect due to their smaller size. Therefore, immunofluorescence is more effective for identifying abnormal NMMHC-IIA localization in neutrophils, and the classification of inclusion bodies also reflects the gene variant locations. In our patient, the variant was found in exon 20, and immunofluorescence revealed type III inclusions. No abnormal inclusions were seen in the neutrophils under light microscopy after Wright's staining of the patient's blood smears, which aligns with existing literature. *MYH9*-RD can also be diagnosed through genetic screening, which includes targeted gene detection (single gene and multigenome detection) and comprehensive genome detection (exome and genome sequencing). The choice of gene detection method depends on the individual's clinical features. Immunofluorescence is advised for diagnosing MYH9 gene variants of uncertain clinical significance (14). Therefore, based on previous research and the diagnostic process of our patient, we recommend routine blood analysis and blood smear microscopy as essential screening methods for *MYH9*-RD. The diagnosis of *MYH9*-RD is based on immunofluorescence and genetic testing, whichcan also aid in predicting patient prognosis (7, 15).

There is a significant link between genotype and clinical phenotype in patients with *MYH9*-RD. Pecci et al. *(*16) were the first to investigate this relationship in 2008, studying 108 *MYH9*-RD patients from 59 families. They found that patients with mutations in the head motor exons were more likely to experience severe thrombocytopenia, a higher number of giant platelets, increased bleeding tendencies, and a greater risk of kidney disease, cataracts, and hearing loss compared to those with mutations in the tail domain of NMMHC-IIA. A later study of the *MYH9*-RD group indicated that the extent of protein conservation loss and the patient's age were more closely related to the phenotype than the specific location of the *MYH9* variant (13, 17). This highlights the intricate relationship between genotype and phenotype, suggesting that patients with mutations in the conserved region of the NMMHC-IIA protein should receive prompt attention and treatment. Consequently, early genetic testing can significantly influence prognosis. There is no notable correlation between elevated liver enzyme levels and genotype (13). A few pathogenic *MYH9* gene variants have been identified in the neck region, with eight publications reporting on these variants as of July 2023. Patients with neck region variants exhibited elevated liver enzymes, hearing loss, mild thrombocytopenia, and FSGS (18–21). Our case displayed these features, indicating that *MYH9*-RD neck gene variants may also lead to multisystem involvement. However, since most reports are based on individual cases, precise data on the proportion of affected organ systems or the incidence of life-threatening complications is still lacking.

The percentage of patients with *MYH9-*RD who experience kidney damage ranges from 30% to 70%. The average age at onset was 27 years. The kidney damage showed proteinuria and microscopic hematuria. However, in *MYH9*-RD patients, proteinuria is a more reliable indicator of glomerular involvement, as hematuria may be caused by thrombocytopenia rather than glomerular lesions. The specific type of renal pathological damage in *MYH9*-RD remains undefined, as the *MYH9* gene can be expressed in glomerular podocytes and mesangial cells, capillary endothelial cells and renal tubules; therefore, *MYH9* variants may cause various types of kidney damage, but due to the fact that patients with the disease often have thrombocytopenia and a high risk of kidney puncture, there are few reports on renal pathological manifestations. Earlier studies (17, 22) have documented findings such as FSGS, interstitial fibrosis, tubular atrophy, and renal microvascular damage. Electron microscopy reveals proliferation without immune deposits, loss of foot processes, and alterations in the glomerular basement membrane. In the majority of nephropathy cases, kidney damage advances to end-stage renal disease. In this instance, the pathological diagnosis for the patient with the *MYH9* neck variant was FSGS. Unlike previous cases, this patient exhibited IgA deposition in the mesangial region, suggesting need for further investigation into prognostic and therapeutic significance of this finding.

The pathogenesis of *MYH9*-RD remains undefined. Both *MYH9* mutants and *MYH9*-deficient cultured podocytes exhibit abnormal podocyte cytoskeleton structure, increased motility and mechanical function (23). Angiotensin II reduces *MYH9* levels in cultured podocytes through Nicotinamide Adenine Dinucleotide Phosphate Oxidase **(**NADPH) oxidase 4 (NOX4) -mediated oxidative stress and transient receptor potential cation channel 6 (TRPC6) activation. Knockdown of *MYH9* or angiotensin II leads to reorganization of podocyte actin cytoskeleton, a decrease in cell adhesion and an increase in albumin permeability (24). Stratifin promotes renal fibrosis via binding with MYH9 in chronic kidney disease (25). These findings may explain the accelerated progression of chronic kidney disease in patients with *MYH9*-RD, potentially triggered by the activation of the renin angiotensin system or other actors affecting *MYH9* expression. Approximately 50% of patients experience chronic elevation of liver enzymes, with levels typically remaining stable over time. Previous studies, along with this case, has not shown any worsening of liver function (18). A liver biopsy revealed mild liver injury in our case. While earlier literature did not provide information on liver pathology, our findings have improved the understanding of liver damage related to *MYH9*-RD.

There is currently no specific treatment for *MYH9*-RD, which primarily focuses on managing symptoms and providing support. Treatments such as glucocorticoids, immunoglobulins, immunosuppressive agents and splenectomy have proven ineffective. Medications that affect platelet function should be avoided. Treatment is not required for patients who do not suffer from bleeding tendencies. Those undergoing elective surgery may benefit from intravenous platelet infusions, thrombopoietin receptor agonists and desmopressin to help prevent bleeding during and after the procedure (26, 27). The most severe complication associated with *MYH9*-RD is nephrosis. Despite its rapid progression and high mortality, there are limited reports of effective clinical treatments. Research indicates that renin angiotensin system blockers can significantly reduce proteinuria in early-stage nephropathy or in cases with minimal progression (24, 28, 29), although treatment failures have also been noted (30). For patients with end-stage renal disease, the only viable treatment options are renal replacement therapies, such as dialysis and kidney transplantation. Kidney involvement is often inked to a poor prognosis (31). In this patient, renal damage was observed early (Table 1 and *Pathological findings of the kidney biopsy*), suggesting a grim outlook. After one year of follow-up, angiotensin system blockers were effective in reducing proteinuria in this case. However, the long-term effectiveness of these medications and the overall prognosis for MYH9-RD will require ongoing monitoring.

In conclusion, *MYH9*-RD is a rare autosomal dominant disorderthat clinicians should be aware of. We recommend routine blood analysis and blood smear microscopy to measure platelet diameter and detect neutrophil inclusions as screening methods for diagnosing *MYH9*-RD. The definitive diagnosis of *MYH9*-RD relies on immunofluorescence detection of abnormal aggregation of NMMHC-IIA in neutrophils, along with genetic testing. To date, there are no specific treatment options for *MYH9*-RD, and further research is needed to elucidate the underlying pathogenesis and and identify potential treatment targets.

## Data Availability

All relevant data is contained within the article: The original contributions presented in the study are included in the article, further inquiries can be directed to the corresponding author.
